# Dynamic Course of *Serratia marcescens* Pulmonic Valve Endocarditis Resulting in Submassive PE and Valve Replacement

**DOI:** 10.1177/2324709618759128

**Published:** 2018-02-26

**Authors:** Chloe Grace Meyer, Thomas Paul Vacek, Amit Bansal, Ravi Gurujal, Analkumar Parikh

**Affiliations:** 1 Wright State University, Dayton, OH, USA

**Keywords:** cardiology, pulmonic valve endocarditis, pulmonary embolism, hypotension, *Serratia marcescens*

## Abstract

This report illustrates a case of a 42-year-old male with a history of intravenous drug abuse who presented with septic shock. Diagnostic studies, including a transthoracic echocardiogram, chest computed tomography angiography, transesophageal echocardiogram, and blood cultures ultimately revealed *Serratia marcescens* pulmonic valve infective endocarditis that was treated with intravenous antibiotics. In addition to the rare form of endocarditis and bacterium involved, this case brings into awareness the dynamic nature of the hospital course that requires vigilance in responding to hypotensive episodes for consideration of pulmonary embolism. Surgical valve replacement was opted for due to such a complication in addition to the large size of the vegetation, 2.5 cm.

## Introduction

Pulmonic valve (PV) infective endocarditis (IE) is extremely rare, accounting for only about 1% to 2.5%^1^ of the total cases of right-sided endocarditis. Because of its rarity, data on PV IE mainly come from a few published case reports, many of which describe only nosocomial acquired infections. It typically occurs in patients with previously damaged valves, ventricular septal defects, or placement of pulmonary catheter.^2^

One of many complications of PV IE are septic emboli, which may present like symptoms of pneumonia. Important complications of which to have constant awareness are embolic events that are estimated to occur in about one third of cases of IE.^3^ However, it is much more likely to occur in right-sided IE, and septic pulmonary emboli are seen in up to 80% of right-sided cases.^4^ Although rare, it is important to consider paradoxical embolism or bilateral endocarditis when evaluating septic emboli.^5^

We describe a patient who presented in septic shock and was subsequently found to have PV IE caused by *Serratia marcescens* with associated bacteremia. Though pulmonic endocarditis is a rare finding in itself among types of endocarditis, the bacterium involved is also very unlikely to infect right-sided valves. What is even more unique about this patient is the recurrent episodes of hypotension with discovery of large PE. It becomes important to be aware that hypotension during a course of pulmonic endocarditis may not be due to sepsis alone, but to large PE burden. Moreover, this process can be dynamic and one should consider the appropriate diagnostic studies at any time.

## Case Description

**Figure 1.**
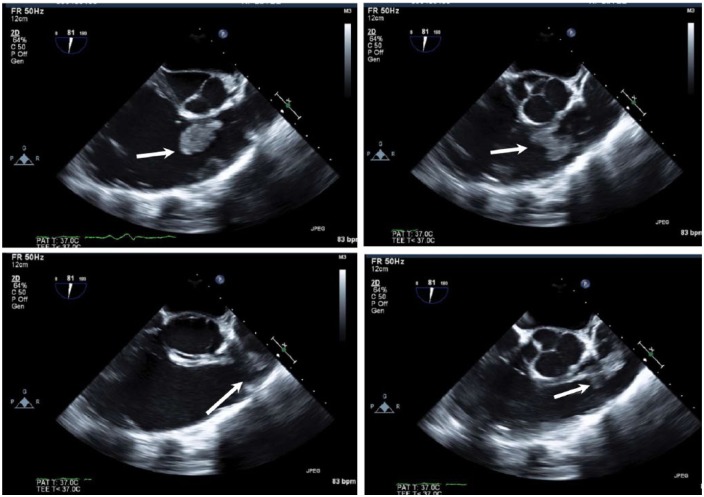
Transesophageal echocardiogram view of the right ventricular outflow tract (RVOT) showing pulmonic valve and hypermobile structure characteristic of vegetation from endocarditis,

**Figure 2.**
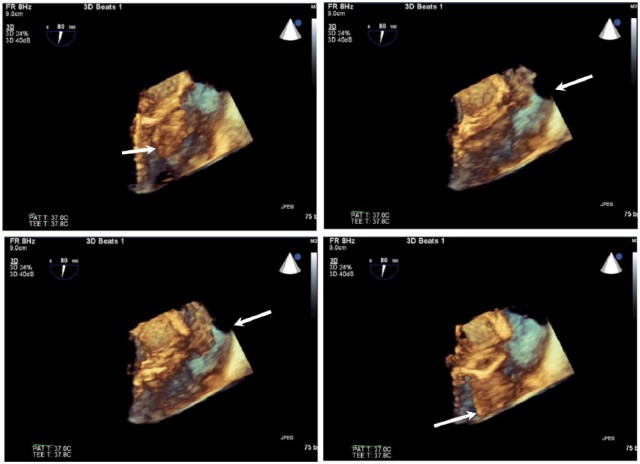
Three-dimensional transesophageal echocardiogram view of the RVOT showing pulmonic valve and hypermobile structure characteristic of vegetation from endocarditis.

**Figure 3.**
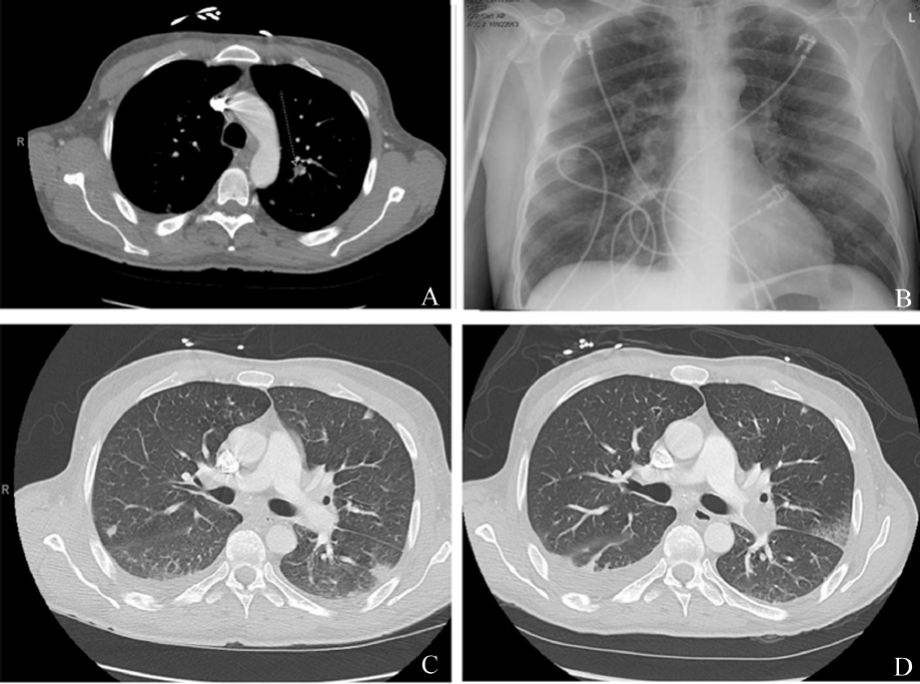
(A) Septic emboli on CT of the chest on the left middle lobe. (B) Septic emboli subtle on the left lung. (C) Initial CT PE protocol negative for PE. (D) Repeat CT PE protocol positive for large left-sided PE.

A 42-year-old Caucasian male with a 1-month history of fevers, chills, and weight loss presented to the emergency department after he developed acute onset of shortness of breath and worsening chills. His past medical history was significant for intravenous drug abuse (IVDU) and hepatitis C. On admission, he was in septic shock with leukocytosis (21.6), lactic acidosis (5.4), thrombocytopenia (35 000), and hyponatremia (126). The patient was placed on broad-spectrum intravenous (IV) antibiotics of vancomycin 1250 mg IV BID and piperacillin-tazobactam 3.375 g IV q8 and vasopressors norepinephrine infusion titrated to mean arterial pressure (MAP) >65 mm Hg after adequate fluid resuscitation. Transthoracic echocardiography (TTE) showed a large vegetation on PV associated with pulmonic insufficiency, and evidence of right ventricle volume overload. Chest computed tomography (CT) showed multiple septic emboli (see Figure 3A-C). Blood cultures grew pan-sensitive *Serratia marcescens*. The repeat blood cultures taken 2 days later showed no growth. After resolution of thrombocytopenia, transesophageal echo (TEE) was performed, which showed a large mobile vegetation attached to the right ventricular outflow tract and PV leaflet, measuring 2.5 × 2.5 cm with multilobulated appearance with several finger-like projections and moderate right ventricle dysfunction (see Figures 1 and 2). After a very complicated course that involved recurrent hypotension with MAP <65 mm Hg in spite of norepinephrine infusion and antibiotic treatment, repeat CT chest showed a new large embolism in the distal left main pulmonary artery with increased infiltrate in the posterior portion of the left upper lobe (see Figure 3D). A repeat TEE showed persistence in vegetation size, now 1.5 cm × 1.5 cm; the patient underwent PV replacement on January 13, 2016. He did well postoperatively and was discharged to nursing home for completion of IV antibiotics ceftriaxone 2 g IV daily for 6 weeks total from date of the initial positive blood cultures.

## Discussion

The main predisposing factors for PV endocarditis in adults are intravenous drug abuse in about 30% of cases and cardiac devices in about 54%.^6^ The patient described here did have a history of IVDU. The pathogenesis for IE in IVDU is not completely elucidated. The general consensus is that the tricuspid and pulmonic valves encounter injected substances before the left-sided valves. This argument, however, fails to address why IE caused by *Serratia* is more commonly left sided even in IVDU.^7^ Nevertheless, it is accepted that injected particles can lead to vasospasm, endothelial damage on the valve, and thrombus formation. This creates the perfect niche for bacteria to grow. Others propose alternative explanations for right-sided endocarditis: different cytokine expression in the right heart^7^ or immune complex deposition after foreign antigens lead to antibody production.^8^ Regardless of the pathophysiology behind right-sided IE, it can be easily differentiated from left-sided IE by the lack of traditional systemic symptoms from septic emboli. Rather, septic emboli from PV IE have been shown to be associated with respiratory symptoms due to the valve’s function and anatomic location.

Etiology of PV endocarditis has most commonly been *Staphylococci* (especially *Staphylococcus aureus*); however, *Serratia marcescens*, a gram-negative bacillus, was isolated. A recent case report and literature review described 3 cases of PV IE with no documented cases due to *Serratia*.^9^ *Serratia* infections are most commonly seen in hospital-acquired urinary tract infections in the elderly.^10^

Diagnosis of PV IE can be challenging because associated murmurs can go undetected and pulmonary presenting symptoms can easily mask another etiology. In addition, the use of the Duke criteria was created based on left-sided IE, and there is currently no specific evidence that it can be applied to PV IE.^11^ When it comes to diagnosis of PV IE, TTE is the gold standard and can detect the majority of cases; however, TEE increases sensitivity of this imaging modality.^12^ Although not yet widely used to diagnose PV IE, cardiac CT with contrast has potential to differentiate between vegetation, thrombus, or tumor if a case is particularly difficult to evaluate on TEE.^12^

Prognostic data of PV IE are limited, though it is generally accepted that size of vegetations and their specific locations both affect prognosis. More is known about prognosis of right-sided IE, and it is generally favorable because its complications are not as hemodynamically severe as left-sided IE. Still though, mortality rates are estimated to be about 5% to 10% for right-sided IE,^13^ and up to 5% to 16% of patients require surgical intervention.^14^ Indication for surgical intervention and its timing in patients with right-sided endocarditis remains unclear. In one case series, there was a marked increase in mortality associated with vegetation greater than 2 cm.^15^ Here we describe a case of PV IE complicated with recurrent pulmonary embolisms from 2.5 cm vegetation requiring PV replacement.

In conclusion, PV IE is a rare entity normally seen in IVDU secondary to *Staphylococcus* bacteremia.^7^ It has potential to be easily misdiagnosed and its dynamic course can easily lead to disaster, such as submassive or massive PE. However, prognosis can be good if prompt IV antibiotics are administered and if there is a low threshold for prompt surgical intervention.
